# Annotations

**Published:** 1909-03-27

**Authors:** 


### Annotations.

### Workhouse Infants.

The publication of the Poor Law Report has provided an occasion for innumerable writers—some of whom are well informed, and some of whom are not—to cry out against everything that is done in Poor-law institutions or in accordance with Poor-law methods. No one denies that in many ways the law may with advantage be amended, and many things might be better managed; but it does not follow that all is as bad as certain sections of the lay Press make out. For example, a popular cry embodies a protest against workhouse lying-in wards, workhouse nurseries, and everything connected with the upbringing of children under the Poor Law. A certain journal—of the kind that takes denunciation for proof—says that the lying-in wards disgrace our civilisation. One instinctively wonders how many of these wards the writer has visited, and if he has also visited the homes of the class of mothers who go to the workhouse. In a metropolitan workhouse are to be found labour rooms with rubber-wheeled beds and every appliance that comfort and propriety demand; an airy ward for mothers and children after parturition; and a well-warmed room for convalescents. Trained midwives are in attendance, and there is a medical man within call by telephone. So good are the conditions under which children are born in these institutions that one of the things which guardians have to beware of is the attempts of married women to get into them for their confinements on the plea that their husbands are out of work. It is true that the conditions are not so good in country workhouses, but even there they are far better than in the places where without them the children would be born. Yet it cannot be denied that a great number of the children born in workhouses die. The main cause of this, however, is to be found in pre-natal conditions. The majority of the workhouse babies are illegitimate, and this means that in almost all cases the mothers have worked too hard, and up to too late a date, in their pregnancy. But it also means that in many cases the mothers have tried to procure abortion. They have failed in their aim, but the child suffers for the attempt. If it is fortunate it is still-born, but there is always the chance that it may struggle on for a few days or weeks of a miserable existence, and when it dies the guardians are blamed! Medical practitioners who have workhouses under their charge, and also have outside practice, are well aware that the proportion of instrument cases is far larger in the lying-in wards of the workhouse than outside, and though it is not a matter on which they can enlarge to the general public, they could easily bring forward many unfortunate infants who are in a physiological sense, even more than in the conventional meaning of the term, base-born. These unhappy infants are suffering for the sins of their fathers and their mothers. And if before long they die, or if they survive as anæmic, backward, or imbecile children, or even attain a maturity of feebleness of body or mind which makes them a burden to the community, let us remember that heredity as well as environment affects life. A generation ago we were too much in danger of forgetting the importance of the latter factor; to-day the tendency, especially of the emotional philanthropist, is to ignore the former.

### A Rest Day for Policemen.

There is sitting at the present time a Select Committee of the House of Commons appointed to consider the advisability of securing a weekly rest day for police constables, the cost of such provision, and other administrative details connected therewith. It appears from evidence given before the Committee that the members of the force, not merely in London, but throughout the kingdom, are strongly in favour of the change. This, of course, is only to be expected; nor is it surprising that they are in favour of receiving the same pay for six days' work as they at present receive for that of seven. On physiological grounds the proposal for this rest day is eminently sound, though we could mention one hardworking section of the community which has to do without it, to wit the medical profession. In favour of the plan have been published the favourable opinions of several leading physicians and surgeons in London, and it is not difficult to agree with what they say. The main difficulty is probably the financial question. A man who joins the police force does so on certain terms which he knows beforehand: his lot may not be in all respects a happy one, but to provide what is practically an advance of some 16 per cent. in his pay is a financial measure to justify which there should be some evidence that the service is unpopular. Be that as it may, we are sorry to see in last week's *Police Review* a bitter attack on Mr. C. T. Dent, the Chief Surgeon to the Metropolitan Force, on account of his published opinions that an extension of the annual holiday would be preferable to a weekly rest day. Mr. Dent's views may or may not be mistaken; they may or may not coincide with the wishes of the constables themselves; or they may possibly be an attempt to obtain for the force a half-loaf when the whole is financially impossible. But to heap abuse upon his head and to suggest to him resignation is a poor way of advancing the cause which the editor of the *Police Review* has at heart. Everyone who knows anything at all about the facts knows that in honesty and sincerity, no less than in the skilled and sympathetic discharge of his official duties, Mr. Dent as an upholder of the interests of the Metropolitan Police stands second to none. It is, unless we err, a fact that about one-seventh of the Force comes upon the sick list annually; and of this number those that are referred to the Chief Surgeon will not, we feel sure, endorse the recriminations of their unofficial Press.

